# Seasonal variations in mortality and clinical indicators in international hemodialysis populations from the MONDO registry

**DOI:** 10.1186/s12882-015-0129-y

**Published:** 2015-08-14

**Authors:** Adrian M. Guinsburg, Len A. Usvyat, Michael Etter, Xiaoqi Xu, Stephan Thijssen, Daniele Marcelli, Bernard Canaud, Cristina Marelli, Claudia Barth, Yuedong Wang, Paola Carioni, Frank M. van der Sande, Peter Kotanko, Jeroen P. Kooman, the Monitoring Dialysis Outcomes (MONDO) consortium

**Affiliations:** Fresenius Latin America, Buenos Aires, Argentina; Fresenius Medical Care, Waltham, MA USA; Renal Research Institute, New York, NY USA; Fresenius Asia Pacific Limited, Hong Kong, Hong Kong; Fresenius Medical Care, Bad Homburg, Germany; Kuratorium für Dialyse und Nierentransplantation e.V., Neu-Isenburg, Germany; University of California - Santa Barbara, Santa Barbara, CA United States; Maastricht University Medical Center, Maastricht, Netherlands; The Mount Sinai Hospital, New York, NY USA; Department of Internal Medicine, Division of Nephrology, University Hospital Maastricht, PO Box 5800, 6202 AZ Maastricht, The Netherlands

**Keywords:** Blood pressure, Global, Hemodialysis, Interdialytic weight gain, Seasonal patterns, Survival

## Abstract

**Background:**

Seasonal mortality differences have been reported in US hemodialysis (HD) patients. Here we examine the effect of seasons on mortality, clinical and laboratory parameters on a global scale.

**Methods:**

Databases from the international Monitoring Dialysis Outcomes (MONDO) consortium were queried to identify patients who received in-center HD for at least 1 year. Clinics were stratified by hemisphere and climate zone (tropical or temperate). We recorded mortality and computed averages of pre-dialysis systolic blood pressure (pre-SBP), interdialytic weight gain (IDWG), serum albumin, and log C-reactive protein (CRP). We explored seasonal effects using cosinor analysis and adjusted linear mixed models globally, and after stratification.

**Results:**

Data from 87,399 patients were included (northern temperate: 63,671; northern tropical: 7,159; southern temperate: 13,917; southern tropical: 2,652 patients). Globally, mortality was highest in winter. Following stratification, mortality was significantly lower in spring and summer compared to winter in temperate, but not in tropical zones. Globally, pre-SBP and IDWG were lower in summer and spring as compared to winter, although less pronounced in tropical zones. Except for southern temperate zone, serum albumin levels were higher in winter. CRP levels were highest in winter.

**Conclusion:**

Significant global seasonal variations in mortality, pre-SBP, IDWG, albumin and CRP were observed. Seasonal variations in mortality were most pronounced in temperate climate zones.

**Electronic supplementary material:**

The online version of this article (doi:10.1186/s12882-015-0129-y) contains supplementary material, which is available to authorized users.

## Background

Biological rhythms underlie multiple physiological processes. This also holds true for dialysis patients in whom disturbances in oscillatory rhythms are of major pathophysiologic importance. Most attention in the dialysis literature has been directed to alterations in ultradian (periods less than 20 h) and circadian (periods between 20 and 28 h) rhythms [1]. However recent studies showed that infradian rhythms (periods longer than 28 h) also have major significance in this population [2–4]. Various clinical and laboratory parameters were found to differ between seasons: in several series both blood pressure and interdialytic weight gain (IDWG) were significantly lower during the summer as compared to the winter [2, 3, 5–7].

Recently we showed in a large cohort of United States (US) hemodialysis (HD) patients that mortality was significantly higher in winter as compared to summer [2]. Moreover IDWG, pre-dialysis systolic blood pressure (pre-SBP) and laboratory parameters such as the neutrophil-lymphocyte ratio (NLR) were also significantly higher in winter. The clinical importance of circannual cycles is further supported by the fact that some of the seasonally variable parameters like IDWG, blood pressure or markers of inflammation are potentially amenable to intervention.

Until now, all studies on seasonal variation in clinical and laboratory parameters in dialysis patients as well as outcome have been performed in temperate climate zones and it is unknown whether these results are applicable in other major climate zones, when analyzed at a global level. The season and/or its associated temperature differences appear to be an important determinant of mortality as demonstrated in the general population in different continents and therefore of importance from a global health perspective [8].

Recently the Monitoring Dialysis Outcomes (MONDO) initiative was initiated with the explicit goal to better understand biological factors and outcomes in chronic dialysis patients. MONDO is an international consortium of dialysis providers from Asia, South America, Europe, and the US [9, 10]. In this global repository, data can be analyzed on a patient level, which allows for the analysis of relevant epidemiological trends in various geographical locations, in patients with diverse ethnicities as well as regions with different practice patterns. The fact that clinics of MONDO partners reside in both Northern and Southern hemispheres and in different climatic zones provides a unique opportunity to study seasonal patterns in mortality and important clinical parameters on a global scale.

In this analysis we studied seasonal variations of indicators with relevance to nutritional, cardiovascular and inflammatory domains, namely IDWG, pre-SBP, serum albumin and C-reactive protein (CRP). The aim of this study was to assess seasonal variations in mortality and selected clinical and laboratory parameters, both globally and after stratification in hemispheres and major climate zones across the globe.

## Methods

At the time of analysis, MONDO consisted of HD databases from Renal Research Institute (RRI) clinics in the US, Fresenius Medical Care (FMC) clinics in Canada, Europe, Asia Pacific (AP) and Latin America (LA), Kuratorium für Dialyse und Nierentransplantation (KfH) clinics in Germany, Imperial College in United Kingdom (UK), Hadassah Medical Center in Israel and Maastricht University Medical Center in The Netherlands. This present study is a retrospective data base analysis; no *a priori* inclusion or exclusion criteria were applied. We queried databases from RRI, FMC Europe (17 countries), FMC AP (9 countries) and FMC LA (5 countries) to identify all HD patients who had in-center HD treatments for at least 1 year between Jan 1, 2000 and Sep 30, 2012. MONDO database includes information on all patients treated in the respective provider network with the data directly extracted from the electronic health record systems. Every individual provider has its own procedures for data cleaning before data end up in the respective provider system. For data collection and analysis, local ethical, compliance, and legal standards are followed. Research conducted by MONDO complies with the Declaration of Helsinki. MONDO partner organizations are responsible for the primary collection and safeguarding of patient data in accordance with all applicable local data protection laws and privacy protection regulations. They also ensure full compliance with laws and regulations regarding the secondary use of data in the context of MONDO. The MONDO data base contains only de-identified data. The New England Institutional Review Board (IRB) has reviewed the claim of exemption for the study and has determined that this research activity is exempt from IRB review Additional file 1 [9, 10].

For each dialysis clinic the major climate zone was identified. We stratified patients into those treated in the Northern and Southern hemisphere respectively, and by tropical and temperate climates. According to standard geographical convention the tropical zone covers the latitudes between 23.5° North and 23.5° South whereas the temperate zone covers the latitudes between 23.5° and 66.5° on the Northern and Southern hemispheres, respectively. No clinics were located in frigid zones (beyond 66.5° North or South, respectively).

In order to correct for geographical region within these zone we used the United Nations (UN) geo-scheme model [11]. We distinguished the following regions: North America, South America, Eastern Asia, Southeastern Asia and Western Asia. Europe was divided into Eastern, Northern, Southern and Western parts. Oceania included Australia and New Zealand.

Seasons were defined based on the calendar months. In the Northern hemisphere, winter was defined as December through February, spring as March through May, summer as June to August, and fall as September to November. In the Southern hemisphere, winter was defined as December through February, spring as March through May, summer as June to August, and fall as September to November.

We computed on a per-patient level and per season mean pre-SBP, IDWG (expressed as percent of post-dialysis weight, IDWG %), log CRP and serum albumin levels. Average values for each season, for each year and for each patient were considered. Only patients with at least one treatment in each of the four seasons were included.

We constructed cosinor models to explore whether cyclical seasonal trends exist in the data.

Cosinor is a set of simple functions that transforms longitudinal data to estimate the cosinor linear model as described in Tong [12]. Methods are given to summarize the mean, amplitude and acrophase, to predict the mean annual outcome value, and to test the coefficients. To determine the impact of seasonality on patient parameters we additionally constructed linear mixed models with random patient-specific intercept for continuous variables with season as a predictor. Linear mixed models were adjusted for treatment year (as categorical variable) and UN geographic region (as categorical variable), hemisphere and climate type (tropical and temperate), to identify whether global changes were not attributed to single treatment years or to a dominant influence of individual regions. Additionally, the models were constructed separately for Northern and Southern hemispheres, and for temperate and tropical climates.

To determine the impact of seasonality on patient mortality we conducted a comparable analysis except that we included all patients irrespective of the number of seasons they were treated in. Cosinor analysis was performed for a visual demonstration of mortality variations between seasons. In addition, the association between seasons and mortality was assessed by a proportional odds model with death as the outcome and the season in which the patient died as the predictor variable with adjustment for treatment year (as categorical variable), UN geographic region (as categorical variable), age, gender, vintage, diabetic status, pre-dialysis systolic blood pressure, interdialytic weight gain, albumin, hemisphere and climate zone. No adjustments for CRP were made, because it was not available in all regions.

An additional analysis was performed after stratification by hemisphere and climate zone. This analysis was performed both unadjusted and after adjustment for treatment year (as categorical variable), UN geographic region (as categorical variable), age, gender, vintage, diabetic status, pre-dialysis systolic blood pressure, interdialytic weight gain and albumin.

Lastly, this analysis was repeated after stratification for climate zone only.

The score test for the proportional odds assumption was not considered significant with p-value of >0.05. The statistical analyses were conducted using SAS version 9.3 (SAS Institute, Cary, NC).

## Results

We studied 87,399 patients (geographical distribution and patient characteristics are shown in Table 1). Mean age of the patients was lower in tropical regions, where serum albumin and blood pressure levels were significantly higher.

**Table 1:** Patient characteristics by hemisphere and climate

Hemisphere	Climate zone	N	Age (years)	% Male	% Diabetic	Pre-SBP (mmHg)	Albumin (g/dL)	log CRP (mg/L)	IDWG (% of post-weight)
Northern	Temperate	63671	59.5 (16.5)	58.1	26.7	140 (20.2)	3.8 (0.5)	1.5 (1.6)	2.5 (1.7)
	Tropical	7159	53.7 (16.6)	63.5	N/A	146.9 (20.2)	3.9 (0.5)	1.3 (1.9)	2.6 (2.3)
	*Total*	*70830*	*59 (16.6)*	*58.5*	*26.7*	*140.8 (20.4)*	*3.8 (0.5)*	*1.5 (1.6)*	*2.6 (1.8)*
Southern	Temperate	13917	57.6 (16.7)	57.3	29.2	134.2 (20.5)	3.6 (0.5)	2 (1.5)	2.8 (2.2)
	Tropical	2652	50.4 (16.3)	59.8	N/A	141.5 (17.5)	3.9 (0.4)	0.2 (1.6)	4.1 (1.7)
	*Total*	*16569*	*56.4 (16.9)*	*57.8*	*29.2*	*135.5 (20.2)*	*3.7 (0.5)*	*1.9 (1.6)*	*3.1 (2.2)*
*Total*		*87399*	*58.4 (16.7)*	*58.4*	*27.2*	*139.5 (20.4)*	*3.8 (0.5)*	*1.6 (1.6)*	*2.6 (1.9)*

Numbers indicate mean (SD) or percent

*CRP* C-reactive protein, *IDWG* interdialytic weight gain, *SBP* systolic blood pressure

### Seasonal variations in mortality

**Fig. 1:**
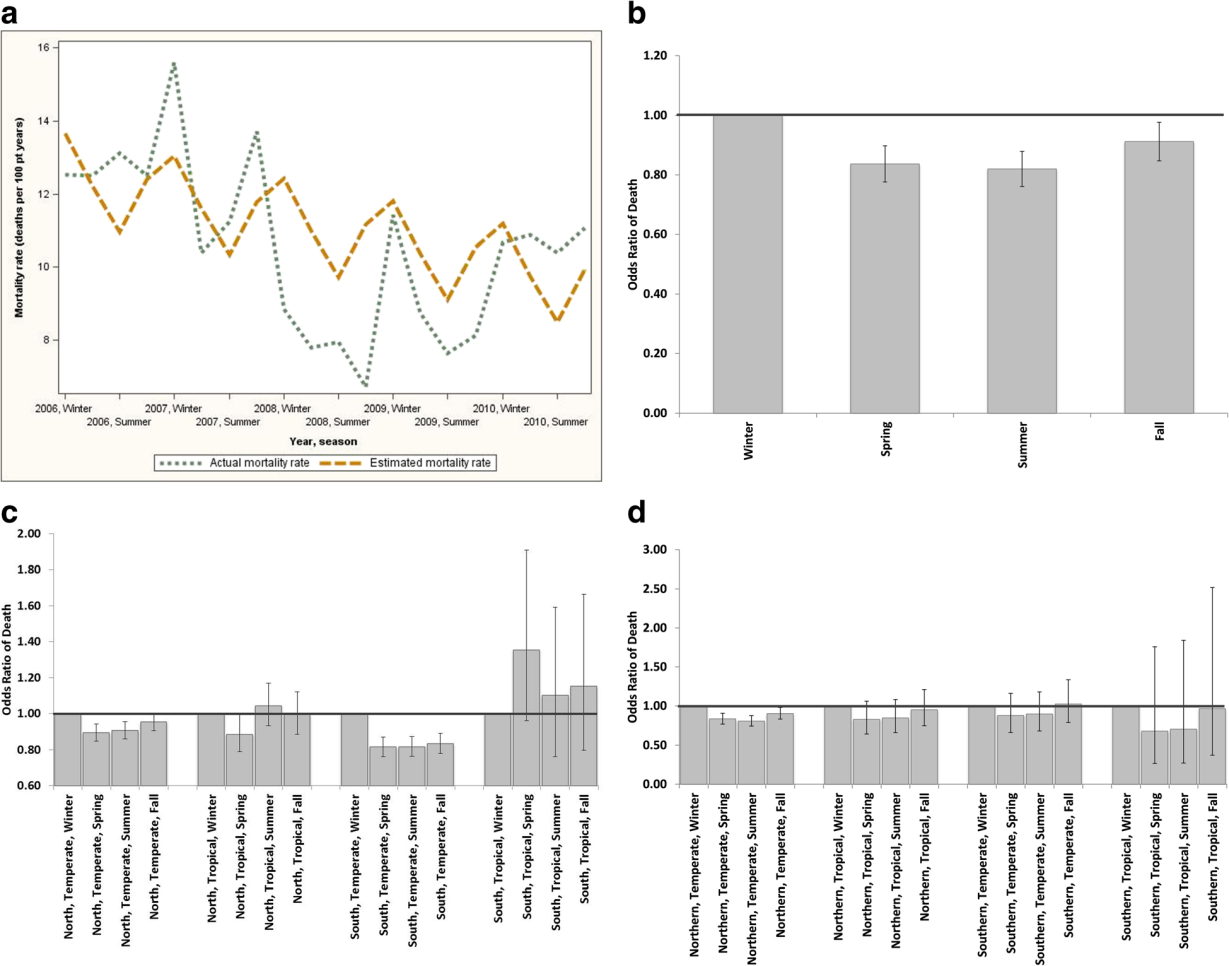
Seasonal variations in mortality rate and odds ratios of death. **a** Actual mortality rate and estimated mortality rate using cosinor analysis (global, unadjusted). **b** Relative risk of death (global, adjusted). **c** Odds ratios (95 % confidence intervals) of death separated per hemisphere, climate zone and season (unadjusted). **d** Odds ratios (95 % confidence intervals) of death separated per hemisphere, climate and season (adjusted)

Cosinor analysis in the overall group indicated clear differences in seasonal mortality (Fig. 1a). These results were substantiated by the proportional odds model and remained significant after adjustment for hemisphere, climate, pre-SBP, albumin, and IDWG (Fig. 1b).

When separated by region and climate zone, unadjusted analysis by the proportional odds model showed that in temperate climates in both hemispheres mortality was lower in spring and summer as compared to winter (fall also lower in Southern temperate zone): North temperate spring odds ratio (OR) 0.89 (0.85–0.94 95 % CI), summer 0.91 (0.86–0.95 95 % CI), fall 0.95 (0.91–1.00 95 % CI). South temperate spring OR 0.82 (0.76–0.97 95 % CI), summer 0.82 (0.76–0.87 95 % CI), fall 0.83 (0.78–0.89 95 % CI). In the tropical climates, mortality was not show significantly different between seasons (with the exception of spring mortality being lower in the Northern tropical climate, (Fig. 1c). After adjustment for multiple confounders, mortality differences only persisted in the Northern temperate zone (Fig. 1d). Lastly, in the analysis pooling both tropical (North and South hemisphere) and temperate (North and South hemispheres), adjusted seasonal variations were only significant in the pooled temperate zone: spring: OR 0.84 (0.78–0.91 95 % CI), summer: OR 0.82 (CI 0.76–0.88) and autumn: OR 0.91 (OR 0.84–0.98). In the pooled tropical zones, despite a tendency to higher mortality in the winter period: seasonal variations did not reach significance: spring OR 0.81 (0.63–1.02 95 % CI), summer: OR 0.84 (CI 0.66–1.07) and autumn: OR 0.96 (OR 0.76–1.21).

### Seasonal variations in pre-SBP and IDWG

**Fig. 2:**
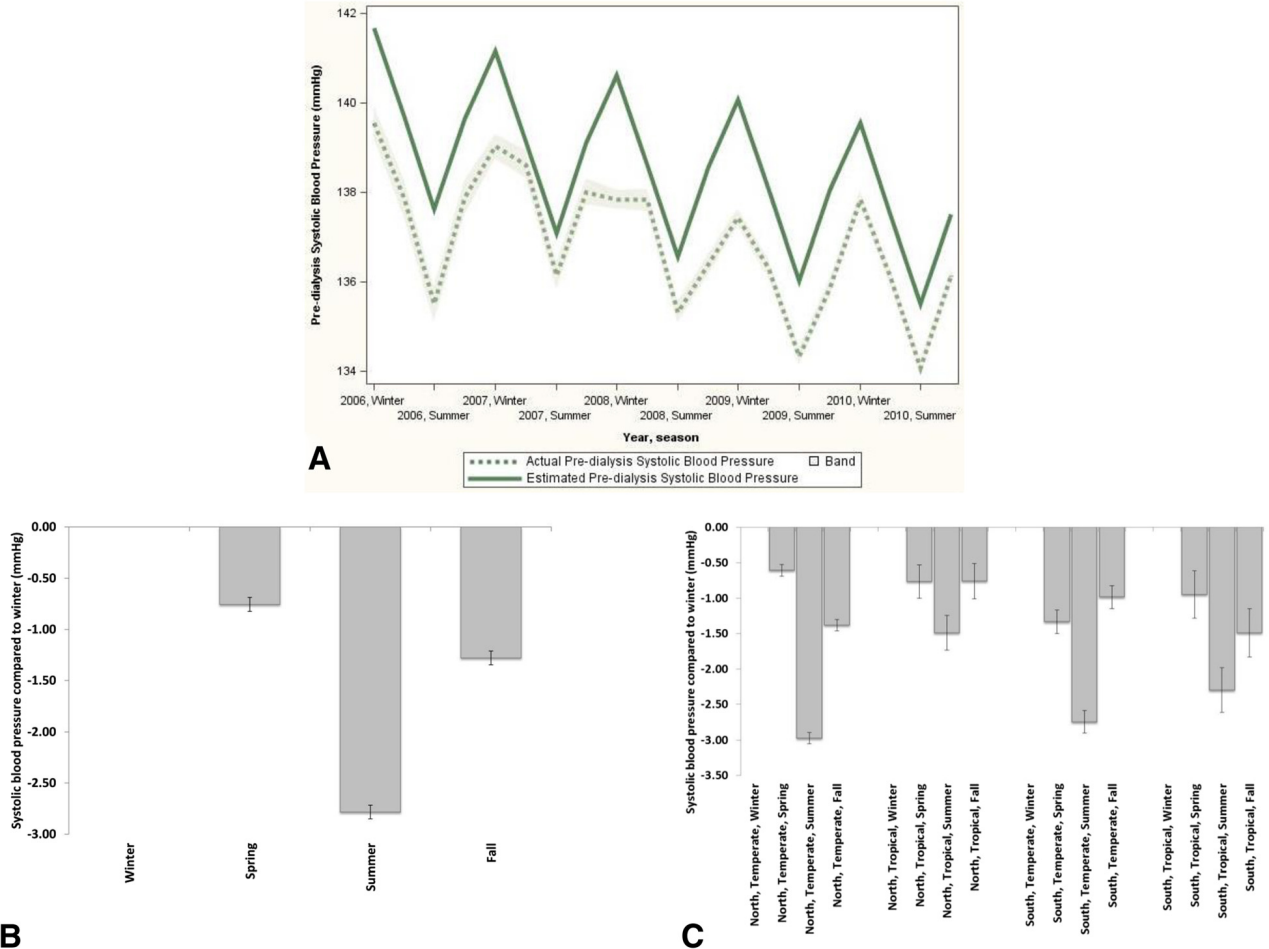
Seasonal trends for pre-dialysis systolic blood presure. **a** Mean actual pre-dialysis systolic blood pressure and estimated pre-dialysis systolic blood pressure using cosinor analysis (global, unadjusted). **b** Difference in pre-dialytic systolic blood pressure (95 % confidence intervals) between seasons with winter as reference (global, adjusted). **c** Difference in pre-dialytic systolic blood pressure (95 % confidence intervals) between seasons with winter as reference, separated by hemisphere and climate zone

Also a clear seasonal trend for pre-SBP was observed using cosinor analysis (Fig. 2a), which was significant also when adjusted for region and climate (Fig. 2b). When separated by region and climate zone, pre-SBP was significantly lower during summer both in tropical and temperate climates in both hemispheres as compared to winter. While significant, the pre-SBP difference between winter and summer appeared less pronounced in tropical climate zones (Fig. 2c).

**Fig. 3:**
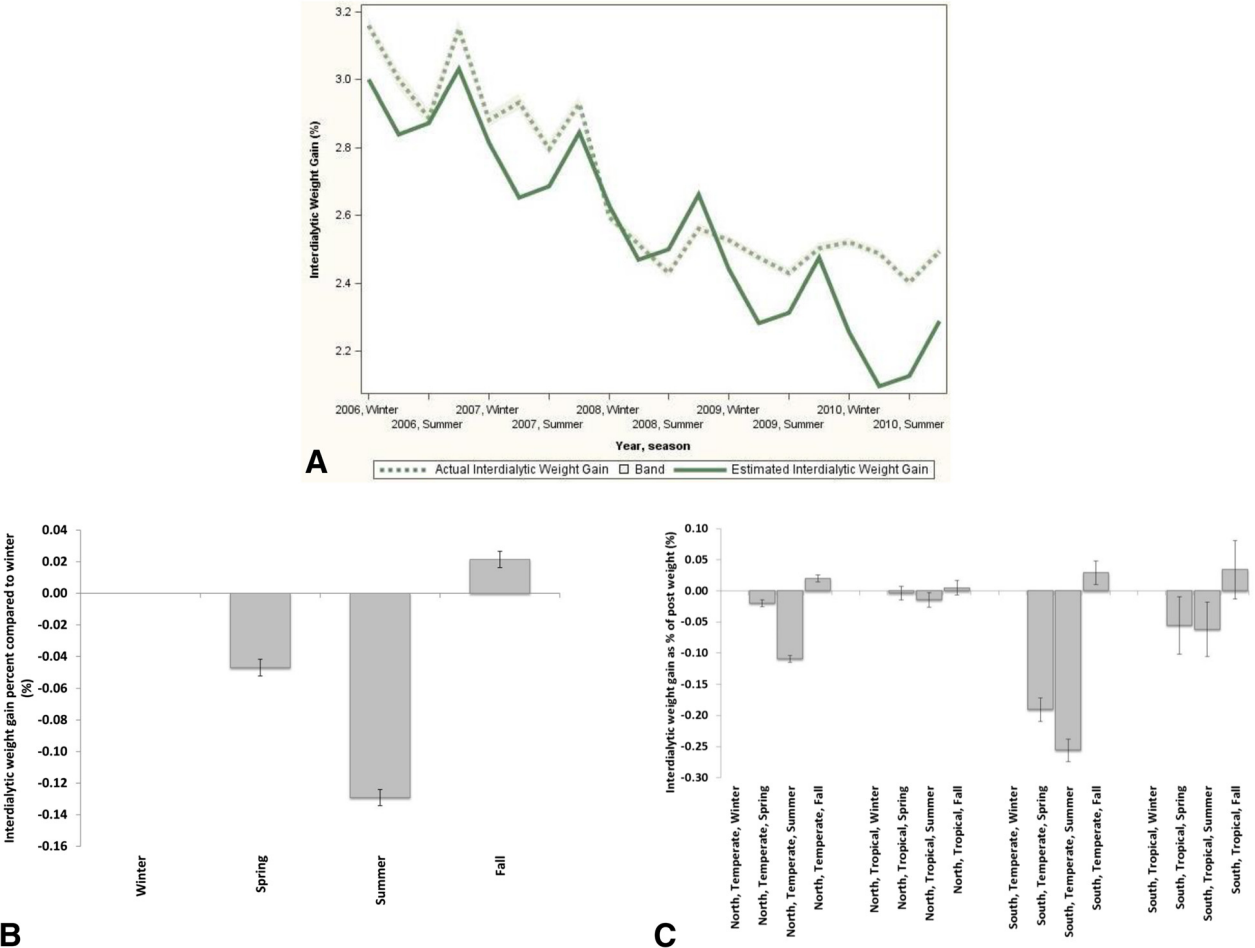
Seasonal trends for interdialytic weight gain. **a** Mean actual interdialytic weight gain percent and estimated interdialytic weight gain using cosinor analysis (global). Interdialytic weight gain is expressed as % of post-dialysis weight. **b** Inter-dialytic weight difference (95 % confidence intervals) between seasons with winter as reference (global, adjusted). Interdialytic weight gain is expressed as % of post-dialysis weight. **c** Inter-dialytic weight gain difference (95 % confidence intervals) between seasons with winter as reference, separated by hemisphere and climate zone. Interdialytic weight gain is expressed as % of post-dialysis weight

Comparable trends were observed for IDWG (Fig. 3a–c), with the lowest values observed during summer, and an apparently smaller seasonal effect for tropical regions.

### Seasonal variations in biochemical parameters

**Fig. 4:**
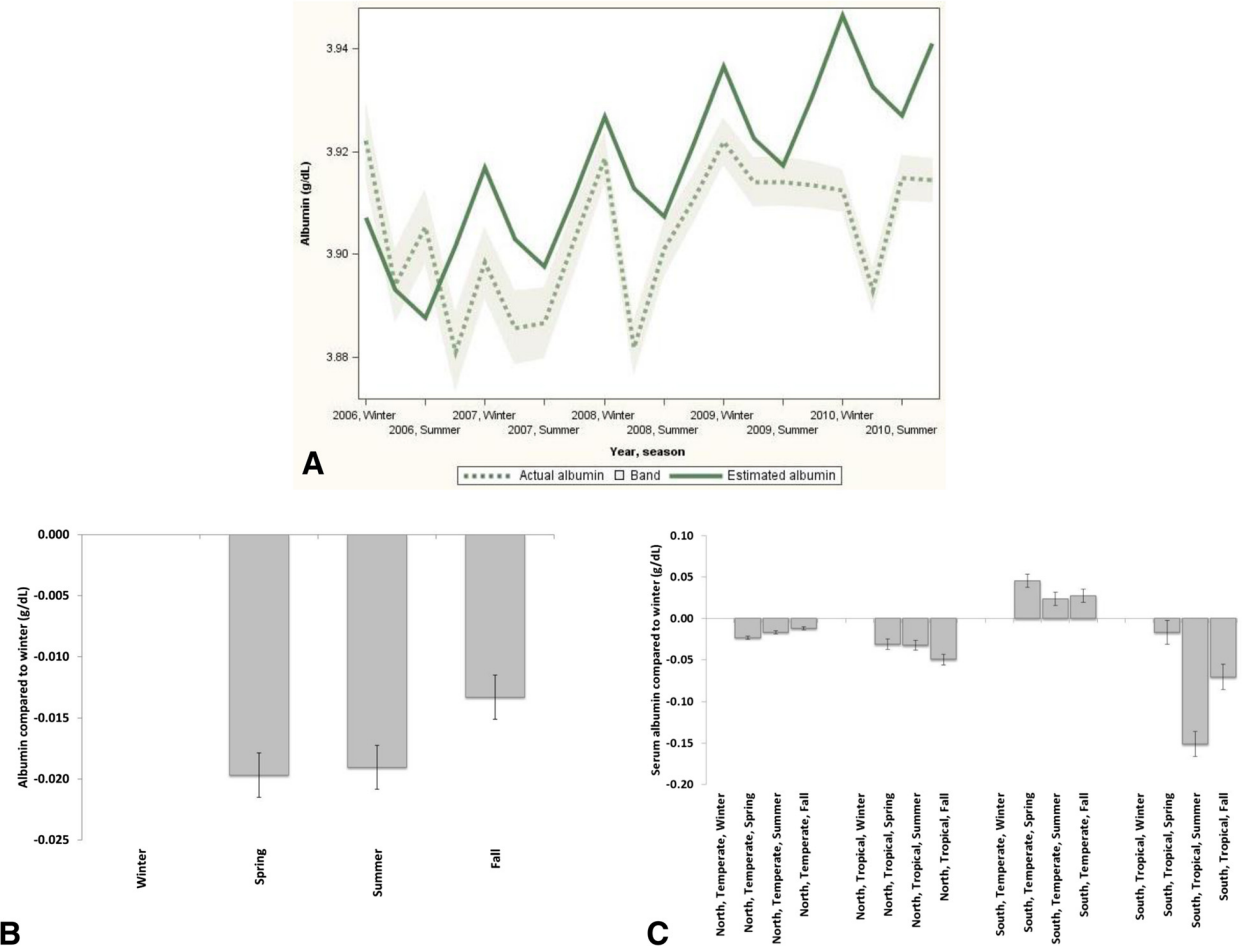
Seasonal trends for serum albumin. **a** Mean actual albumin (95 % CI) and estimated albumin using cosinor analysis (global, unadjusted). **b** Serum albumin difference (95 % confidence intervals) between seasons with winter as reference (global, adjusted). **c** Serum albumin difference (95 % confidence intervals) between seasons with winter as reference, separated by hemisphere and climate zone

In general, serum albumin levels were higher during the winter as compared to other seasons (Fig. 4a and b). However, while albumin levels were significantly higher during winter in tropical climates in both hemispheres and in the North temperate climate (Fig. 4c), they were lower during winter in South temperate climate.

**Fig. 5:**
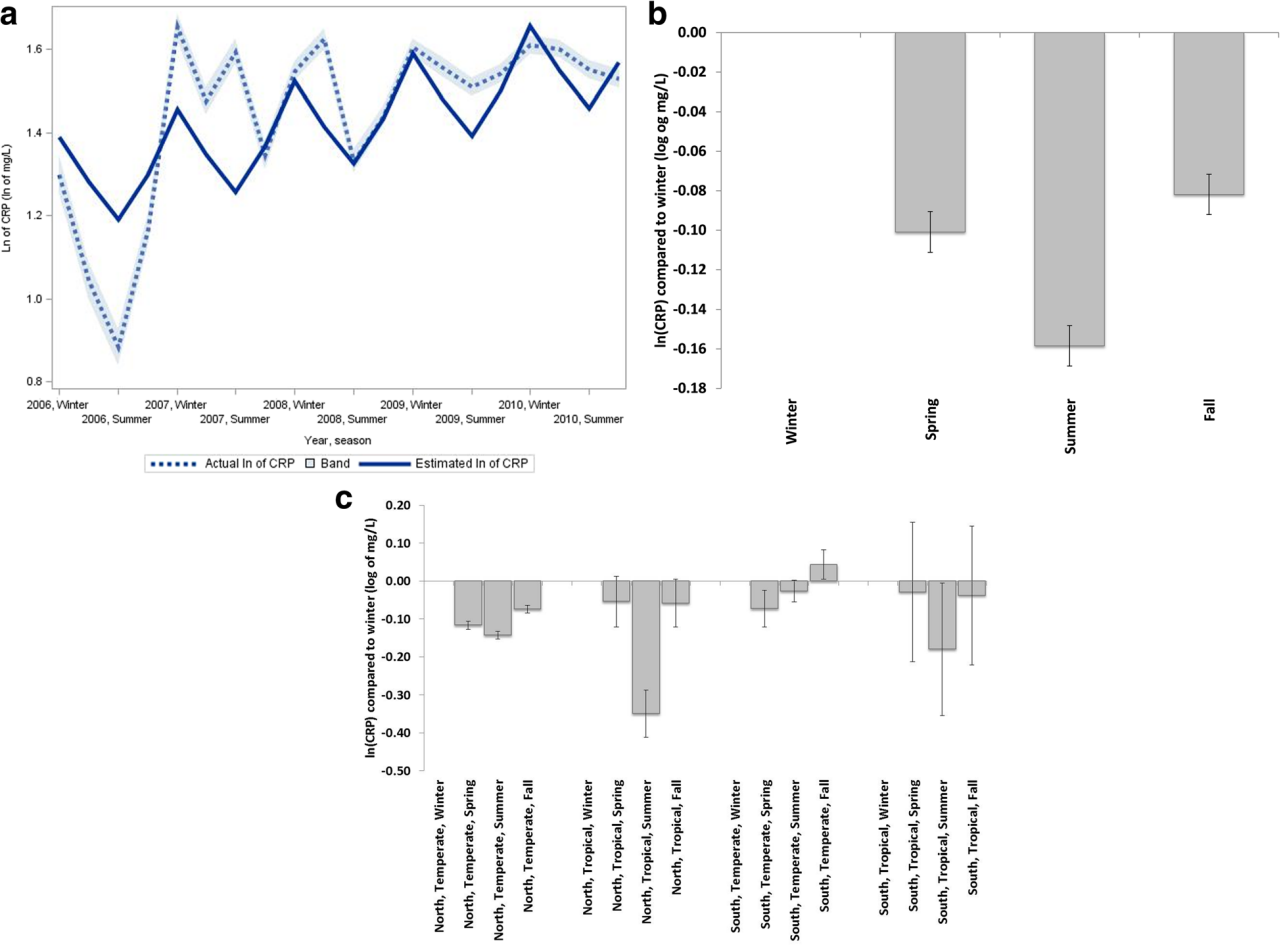
Seasonal trends for log transformed C-reactive protein, ln (CRP). **a** Mean actual log (ln) of CRP (95 % CI) and estimated log of CRP using cosinor analysis (global, unadjusted). **b** log (ln) C-reactive protein difference (95 % confidence intervals) between seasons with winter as reference (global, adjusted). **c** log (ln) of C-reactive protein difference (95 % confidence intervals) between seasons with winter as reference, separated by region and climate zone

In contrast, log-transformed CRP levels were highest in winter (Fig. 5a and b). When analyzed separately by hemisphere/climate region, CRP levels were significantly lower in temperate climates in both hemispheres in summer and spring compared to winter; fall levels were less conclusive (Fig. 4a). In tropical climates summer CRP levels were also significantly lower than in winter with patients manifesting significantly lower CRP levels in this season compared to those in temperate climates.

## Discussion

This study assessed seasonal variations in mortality and important clinical and laboratory parameters in dialysis patients in a large cohort of dialysis patients. This study adds to the current body of knowledge by exploring for the first time seasonal trends in dialysis patients on a global basis, including countries of both Northern and Southern hemispheres and of temperate and tropical zones. The present study expands our previous observation of major seasonal differences in mortality in a large cohort of chronic HD patients from the US [2].

At a global level, mortality was highest in winter and lowest in spring and summer. In our previously studied US cohort, mortality differences between seasons lost statistical significance when adjusting for seasonally variable risk factors such as blood pressure, albumin and inter-dialytic weight gain. However, the present study had far greater statistical power and clearly indicates that at a global level seasonal differences remained significant after adjustment for seasonally variable risk factors. Most likely, other factors, such as underlying diseases, influence seasonal mortality but are not reflected in the limited palette of clinical and laboratory parameters included in the present study.

Following stratification and adjustment, seasonal variations in mortality appeared more pronounced in northern temperate zones, whereas the variations in mortality in tropical zones did not reach significance, although the tendency to a higher winter mortality was also observed in tropical regions. Admittedly, patient characteristics differed between zones, but also after adjustment for multiple confounders, seasonal variations in mortality were only significant in the temperate zones, although this.

Several general hypotheses can be put forward to explain the apparent differences between climate zones. Firstly, in tropical regions seasons are often more characterized by differences in precipitation and not by classical four-season’s patterns, with accompanying pronounced differences in seasonal temperature. Secondly, seasonal changes in direct sunlight hours which might affect vitamin D levels are less pronounced in tropical as compared to temperate regions. Thirdly, both low winter temperatures and high summer temperatures are also associated with mortality in the general population [13, 14, 11], and the latter effect may be more pronounced in tropical regions.

Our findings are in line with a recent study in general population which showed that seasonal differences in mortality in a US population were lowest in Miami, which has a tropical climate [15]. On the other hand despite much lower seasonal temperature differences, the variation in cardiac mortality was comparable between Honolulu and US cities located in temperate regions [16]. Lastly, although the number of patients in each different zones was substantial, the smaller number of patients in tropical as compared to temperate regions might affect the margin of error in the confidence intervals of mortality differences between seasons, although the pooled tropical zones still contained 9811 patients. Still, the potential differences in seasonal mortality between the various climate zones should be interpreted with caution.

Even in the general population comparative studies of seasonal mortality in different global geographical zones are scarce. In the ISOTHURM study mortality was higher in winter periods, with more pronounced differences for temperate as compared to tropical latitudes [8], showing a significant relation between ambient temperature and mortality. In contrast to the ISOTHURM study, ambient outdoor and indoor temperatures were not available in the present study. Therefore, we cannot make firm statements about the relation between ambient temperature and mortality [8].

The pathophysiological mechanisms underlying the higher winter mortality rates in dialysis patients remain hypothetical. In our previous US study, we found that cardiovascular events, the most important cause of mortality in dialysis patients, contributed predominantly to the higher mortality in winter [2], an observation in agreement with findings in the general population [8]. Both fluid overload, as indicated by higher IDWG, increased peripheral vasoconstriction and release of pro-thrombotic factors [17], as well as a higher incidence of infectious complications and subsequent cardiovascular complications [18], could be responsible for the observed seasonal mortality differences. A recent study in the general population showed that influenza epidemics accounted for 18 % of the seasonal variation in cardiac mortality [16]. Regrettably we were not able to determine the exact causes of death in the present cohort. Also no data on vaccinations are available in our cohort.

Next to the differences in mortality, pre-SBP and IDWG showed seasonal changes in all geographical zones. These were observed both in temperate and - less pronounced - tropical zones. Higher IDWG and pre-SBP have been observed in winter periods in dialysis patients dwelling in Northern [2, 3, 5] and Southern temperate zones [6]. To the best of our knowledge only a single study from Brazil addressed seasonal differences in blood pressure in a tropical climate [19]. This study reported higher diastolic and mean blood pressure in winter, but no difference in IDWG between seasons. The mechanisms behind seasonal differences in SBP have been discussed previously and possibly include volume-related factors, differences in vascular tone or differences in vitamin D_3_ levels [2, 3, 5, 6, 20]. The lower IDWG in summer could be related to increased perspiration and/or differences in fluid intake.

Seasonal differences in serum albumin and CRP are somewhat more difficult to interpret. At a global level, CRP levels were lower in the summer period in all geographical zones, which may point to a lower risk of infectious episodes in summer periods [8]. The lower CRP levels are in agreement with the lower neutrophil-to-lymphocyte ratio (NLR) in summer which we observed in our previous study [2]. A recent study from Dopico et al. [21] showed a seasonal expression profile for more than 4,000 protein-coding mRNAs in white blood cells and adipose tissue with inverted patterns observed between Europe and Oceania. They also found increased levels of soluble IL-6 receptor and CRP during European winter, risk biomarkers for cardiovascular and autoimmune diseases with peak in winter. With the exception of southern temperate regions, serum albumin levels were also lower in the summer period. While these results contrast with our previous study in US HD patients [2], in which no seasonal differences in serum albumin levels were observed, they are in agreement with findings from a Japanese dialysis population [7]. The fact that serum albumin levels, a strong marker of survival, were lower in the season when the relative risk of death, as well as CRP levels are also at their nadir appears contradictory. The interpretation of serum albumin levels is complicated since albumin concentrations are the compound resultant of inflammation, nutrition, and fluid status. It is possible that a higher dietary intake during winter [7] explains why serum albumin levels are higher in this season. Data from other studies are similarly inconclusive. In the study by Cheung in North American patients [3], protein intake appeared to be highest in early spring whereas serum albumin levels were highest in October. From the present results, we are not able to explain the discrepant results in Southern temperate zones as data on dietary intake are not available in our cohort. It is important to realize that these observations only imply that in the population studied albumin levels are higher during winter, and not that the relation between mortality and lower serum albumin levels would be different in the MONDO population. In a previous study from the MONDO cohort, we showed significant declining temporal trends in serum albumin levels before death [22].

The findings of the present study may have clinical implications. Both pre-SBP and IDWG are related to outcome in dialysis patients [23, 24]. Higher IDWG can result in hypertension, left ventricular hypertrophy, cardiac failure, and pulmonary congestion [25]. Although data on this aspect are scarce, a recent study in peritoneal dialysis patients suggests that intensive nursing care (including education on fluid management, home blood pressure measurements and intensified antihypertensive treatment) could reduce the seasonal differences in blood pressure [26]. Given the relatively large effect of influenza epidemics on cardiac mortality in the general population, special attention to vaccination for influenza and possibly pneumococcus in this vulnerable population appears warranted [27]. Also cholecalciferol supplementation particularly during sun-deprived seasons in temperate zones was shown to have a beneficial effect on inflammatory and cardiac parameters [28]. However, future studies should address whether the increased mortality during winter in temperate zones is amendable to intervention. Finally, seasonal differences in mortality may be of importance in the design of clinical trials and the interpretation of their results.

Our study has various limitations. Aside from to the absence of data on ambient temperatures, the division in geographical zones and regions is only a rough proxy of climatological and geographical differences. Therefore differences between smaller geographical regions are not captured in this study. In addition, despite the fact that the MONDO consortium includes countries within different geographical zones and the results presented here draw on the currently most diverse international patient-level hemodialysis data, they certainly do not reflect the global hemodialysis population and many important countries with large dialysis populations are currently missing or underrepresented. For example, neither dialysis clinics from Japan nor the large corporate US hemodialysis providers are members of the MONDO initiative. Moreover, because of data logistics and availability some MONDO data, in particular data from a large German provider and some smaller academic providers, were not included in the current analysis. For this reason, the data came primarily from three large and one medium-sized corporate dialysis provider. Despite that limitation we feel that geographical coverage and patient diversity are sufficient to support our conclusions. Nevertheless, it would be interesting to conduct a comparable study including public and academic dialysis centers. Another limitation is that we could not report the mortality rates by causes of death. Also, within each geographical area there is likely to be variation in mortality rates between providers. However, strict provider compliance regulations prevent the publication of their mortality rates. The cosinor method estimates parameters representing cyclic events by computing a least squares fit of a cosine function. While widely and successfully used in the analysis of seasonal data, the cosine method has certain shortcomings; for example, the parameter estimates may be biased if the raw data substantially depart from a cosine function. However, the fact that visual inspection of the data indicated seasonal patterns motivated and reassured us to use the cosinor method.

## Conclusion

In this large global dialysis study we observed significant variations in seasonal mortality, with highest value in winter. These differences appeared more pronounced in temperate as compared to tropical climate zones. Also, at a global level, marked seasonal patterns of key clinical and laboratory parameters, were observed with higher levels of pre-SBP, IDWG, serum albumin and CRP levels in winter as compared to summer.

## Additional file

Additional file 1:
**The MONitoring Dialysis Outcomes (MONDO) initiative.** (DOCX 39 kb)

## Abbreviations

AP: Asia Pacific
CRP: C-reactive protein
FMC: Fresenius Medical Care
HD: Hemodialysis
IDWG: Interdialytic weight gain
KfH: Kuratorium für Dialyse und Nierentransplantation clinics
LA: Latin America
MONDO: Monitoring Dialysis Outcomes consortium
NLR: Neutrophil-lymphocyte ratio
OR: Odds ratio
pre-SBP: Pre-dialysis systolic blood pressure
RRI: Renal Research Institute clinics
UK: United Kingdom
UN: United Nations
US: United States
