# Supplementary material for: Seasonal variations in mortality and clinical indicators in international hemodialysis populations from the MONDO registry
Source: BMC Nephrol. 2015 Aug 14;16:139. doi: 10.1186/s12882-015-0129-y (PMC4542126; doi:10.1186/s12882-015-0129-y)
Supplement: Additional file 1: — The MONitoring Dialysis Outcomes (MONDO) initiative. (DOCX 39 kb) [file 12882_2015_129_MOESM1_ESM.docx]

**The MONitoring Dialysis Outcomes (MONDO) initiative**

The MONDO initiative was founded in 2009 as a joint initiative of several dialysis providers. MONDO subsequently developed into a consortium in which a variety of academic and nonacademic institutions from around the world work together on research projects to analyze primary clinical databases of dialysis patients[[1](#_ENREF_1)].

The instigating event back in 2008 was the insight that in a small cohort of Black and White US dialysis patients treated in hemodialysis facilities of the Renal Research Institute (then a joint-venture partnership between Beth Israel Hospital New York and Fresenius Medical Care North America) death was preceded by typical events, such as decline in blood pressure and albumin[[2](#_ENREF_2)].

The authors of that paper were intrigued by the question if their observations were specific to US patients only or may actually indicate more general biological phenomena. However, back then there was no international treatment-level data base available to explore that question further. The authors of that paper, Claudia Barth (then Chief Medical Officer at the KuratoriumfürHeimdialyse, the largest German dialysis provider), Michael Etter (Chief Medical Officer for Fresenius Medical Care Asia Pacific) and Emanuele Gatti (then CEO Fresenius Medical Care Europe, Middle East, Africa, Latin America) conceived the idea to found MONDO with the explicit goal to describe biological phenomena in dialysis patients which appear to be operative irrespective of the region of the world, gender, race, dialysis practice pattern, and facility ownership.

MONDO soon included providers, large and medium sized corporate ones (KuratoriumfürHeimdialyse, Fresenius Europe, Middle East, Africa, Latin America, RRI in the US), and smaller academic ones (Maastricht University, The Netherlands; Hadassah University Jerusalem; Imperial College London, UK; Curitiba University, Brazil). Over the years more providers and data owners have joined (BRAZPD Brazil; NephroSolutions, Germany; Catharina Ziekenhuis Eindhoven, The Netherlands; Soba University Hospital, Sudan); and additional partners are expected to follow.

Methodology

MONDO can be viewed as a global registry composed of datasets from several primary databases[[3](#_ENREF_3)]. It is a research collaboration that explores established clinical databases of dialysis patients across national, regional and provider borders and constitutes a framework for a variety of observational study designs. MONDO encompasses patients of diverse ethnic backgrounds who are treated in different medical systems and under different reimbursement policies. To facilitate joint analyses across de-identified datasets from different consortium members, primary datasets are converted into a uniform data structure for MONDO. The resultant MONDO analytical files, which together constitute a virtual MONDO ‘data bank', are either kept locally for analysis if requested by the provider or analyzed centrally at the Renal Research Institute.

Funding

Funding for the primary databases including data conversion into the MONDO data structure is provided by each consortium member. Expenses related to data analysis and statistical support, if not performed by the members themselves, are covered by the Renal Research Institute. Participation in joint meetings and other administrative costs are the responsibility of each member.

Ethical Standards, Patient Confidentiality and Data Protection

Patient participation follows the standards of the Declaration of Helsinki and Good Epidemiological Practice in all organizations. All organizations are responsible for the primary collection and safeguarding of patient data in accordance with all local data protection laws and privacy protection regulations. They are also responsible for ensuring compliance with laws and regulations regarding the secondary use of data in the context of MONDO and obtaining informed consent.
Every MONDO analysis is made by transforming de-identified primary data into a uniform MONDO data structure. In most instances, the anonymized patient data are analyzed by RRI. KfH does not share data but uses jointly developed statistical analysis codes and analyzes data locally in accordance with provider rules, their patients' written consent and local privacy protection laws.

Data availability and subjects

The first converted datasets covered patients treated between January 2000 and December 2010. After that, data updates are scheduled at regular intervals.

Nowadays, the following providers have converted datasets for MONDO: the RRI (USA), Fresenius Medical Care (FMC) Europe, Latin America and Asia-Pacific, the KuratoriumfürDialyse und Nierentransplantatione.V. (*Curatorium for Dialysis and Kidney Transplantation*; KfH, Germany), Catharina Hospital in Eindhoven (The Netherlands), and Imperial College London (UK).

The MONDO database currently contains more than 200,000 chronic dialysis patients from 37 countries on 5 continents: Europe and Middle East, 23 countries; Latin America, 5 countries; Asia-Pacific, 8 countries including Australia and New Zealand, and USA.
Overall patients' average age is 61.8 years; there are 41% women, and 28% are diabetic. There are approximately 43 million hemodialysis and 3 million peritoneal dialysis treatments in the database along with associated clinical and laboratory parameters. Longitudinal data are also available from over 1,500 pediatric patients aged 17 years and younger[3].

Statistical Approach to MONDO databank

MONDO provides a platform for a variety of observational studies. Statistical analysis plans are developed collaboratively for each scientific project under the statistical oversight of the Department of Statistics and Applied Probability, University of California Santa Barbara, California, USA.

Which methods to use depends on the specific scientific questions, but, in general, they are required to deal with repeated measurements over time[[4](#_ENREF_4), [5](#_ENREF_5)].

Since there are a large number of longitudinal measurements and none of them can fully predict the outcome, new statistical methods need to be developed to reduce dimensionality of high-dimensional longitudinal data that are also subject to informative censoring due to death. The large volume of data also poses computational challenges. For example, in a recent publication, the characteristic dynamics before death for interdialytic weight gain, systolic blood pressure, serum albumin and C-reactive protein were investigated[[6](#_ENREF_6)]. To estimate the mean functions and the trajectories of these variables, quintic spline models were fitted to partial conditional means. So far, the mean function for each variable was studied separately. Combining all variables with a goal towards building an alarm system that alerts the clinician to incipient deterioration of their patients' condition remains an important research topic.
As well as the previous mentioned study, some others recent publications from MONDO databank can be found elsewhere showing different data analysis and statistical approaches according to specific scientific questions[[7-10](#_ENREF_7)]. Therefore, for the ultimate goal of improving outcome in dialysis patients, the MONDO initiative provides big challenges and ample opportunities for statistical method development.

Scope of the project

The scope of MONDO is broad and includes populations from areas which are not often included in international studies. This increases generalizability of results and the ability to detect rare events. However, it should be noted that primary databases are often not representative of their national source population. Therefore, prevalence and incidence results in the MONDO cohort should be interpreted with caution.

Conclusion

In summary, MONDO represents a dataset which holds great promise for a much more detailed understanding of the clinical course of patients on dialysis. It may serve as the foundation for the development of intelligent alert systems that can improve patient care worldwide.

**References**

1. Usvyat LA, Haviv YS, Etter M, Kooman J, Marcelli D, Marelli C, Power A, Toffelmire T, Wang Y, Kotanko P: **The MONitoring Dialysis Outcomes (MONDO) initiative**. *Blood purification* 2013, **35**(1-3):37-48.

2. Kotanko P, Thijssen S, Usvyat L, Tashman A, Kruse A, Huber C, Levin NW: **Temporal evolution of clinical parameters before death in dialysis patients: a new concept**. *Blood purification* 2009, **27**(1):38-47.

3. von Gersdorff GD, Usvyat L, Marcelli D, Grassmann A, Marelli C, Etter M, Kooman JP, Power A, Toffelmire T, Haviv YS *et al*: **Monitoring dialysis outcomes across the world--the MONDO Global Database Consortium**. *Blood purification* 2013, **36**(3-4):165-172.

4. Diggle P LK-Y, Zeger SL: **Analysis of Longitudinal Data**: Oxford; 1994.

5. Ramsay JO SB: **Functional Data Analysis**, 2nd edn. New York: Springer; 2005.

6. Usvyat LA, Barth C, Bayh I, Etter M, von Gersdorff GD, Grassmann A, Guinsburg AM, Lam M, Marcelli D, Marelli C *et al*: **Interdialytic weight gain, systolic blood pressure, serum albumin, and C-reactive protein levels change in chronic dialysis patients prior to death**. *Kidney international* 2013, **84**(1):149-157.

7. Broers NJ, Usvyat LA, Marcelli D, Bayh I, Scatizzi L, Canaud B, van der Sande FM, Kotanko P, Moissl U, Kooman JP *et al*: **Season affects body composition and estimation of fluid overload in haemodialysis patients: variations in body composition; a survey from the European MONDO database**. *Nephrology, dialysis, transplantation : official publication of the European Dialysis and Transplant Association - European Renal Association* 2015, **30**(4):676-681.

8. Calice-Silva V, Hussein R, Yousif D, Zhang H, Usvyat L, Campos LG, von Gersdorff G, Schaller M, Marcelli D, Grassman A *et al*: **Associations between global population health indicators and dialysis variables in the Monitoring Dialysis Outcomes (MONDO) consortium**. *Blood purification* 2015, **39**(1-3):125-136.

9. Marcelli D, Usvyat LA, Kotanko P, Bayh I, Canaud B, Etter M, Gatti E, Grassmann A, Wang Y, Marelli C *et al*: **Body Composition and Survival in Dialysis Patients: Results from an International Cohort Study**. *Clinical journal of the American Society of Nephrology : CJASN* 2015, **10**(7):1192-1200.

10. Malhotra R, Marcelli D, von Gersdorff G, Grassmann A, Schaller M, Bayh I, Scatizzi L, Etter M, Guinsburg A, Barth C *et al*: **Relationship of Neutrophil-to-Lymphocyte Ratio and Serum Albumin Levels with C-Reactive Protein in Hemodialysis Patients: Results from 2 International Cohort Studies**. *Nephron* 2015.
